# Variation in oxytocin receptor DNA methylation in female chickens are associated with age, genetics, laying intensity, and housing conditions

**DOI:** 10.1016/j.psj.2026.107667

**Published:** 2026-08-20

**Authors:** Claudia Klein, Steffen Weigend, Christian Reimer

**Affiliations:** Institute for Farm Animal Genetics, Friedrich-Loeffler-Institut, Mariensee, Germany

**Keywords:** Layer chicken, Methylation, Genetics, Housing, Laying intensity

## Abstract

Animal welfare monitoring is rapidly becoming a critical concern, with an ongoing search for biomarkers. DNA methylation has the potential to serve as such a biomarker, as environment and genotype can interact to shape DNA methylation. We tested the hypothesis, whether genetically distinct laying hens display divergent methylation patterns at selected genes relevant for behavior in response to housing conditions. The aims of the present study were therefore to determine the effect of age (two-day-old chicks versus one-year-old laying hens), genetics (brown versus white laying hens), laying intensity (high versus low), and the effect of housing conditions (layer cage versus small group housing) on the methylation levels of selected genes implicated in behavior in chicken. DNA methylation at the OXT, OXTR, ESR1, HTR1A, HTR2A, and HTR3A gene were determined using direct bisulfite-PCR sequencing, with OXTR being the only gene displaying consistent effects. The greatest effect of age on methylation was evident for OXTR, with adult animals displaying significantly higher methylation than 2-day old chicks. While methylation levels for OXTR did not differ between chicks, significant differences in methylation levels were detected for OXTR when comparing samples from adult animals. For the majority of CpG sites, an interactive effect of laying intensity x housing and genetics x housing on OXTR DNA methylation was evident. When evaluating OXTR methylation status separated by housing condition, a clear difference between layer lines was revealed. While BLA (brown, high laying intensity) animals exhibited consistent methylation levels independent of the housing condition, WLA (white, high laying intensity), G11 (white, low laying intensity) and L68 (brown, low laying intensity) animals displayed differences in OXTR methylation depending on the housing condition. In summary, age, genetics, laying intensity, and housing conditions are associated with differences in DNA methylation of the oxytocin receptor in female chicken, suggesting that OXTR methylation may represent a candidate epigenetic biomarker linking genetics, environment, and laying performance. Future studies will have to determine its correlation with animal welfare status.

## Introduction

Intensive poultry production housing conditions have the potential to affect the behavior of chickens. Housing layer hens in conventional "battery" cages is therefore prohibited in Germany and across the European Union due to animal welfare concerns. Laying hens in cages show several characteristic behavior problems that stem from frustrated motivation and restricted movement, with feather pecking, abnormal dustbathing, and nesting frustration being the most prominent (Baxter, 1994). In contrast, housing hens in small groups allows for more natural behavior, with age, laying intensity, and genetics having the potential to shape welfare-relevant phenotypes (Grethen et al., 2023). Animal welfare monitoring is rapidly becoming a critical concern, and there is an ongoing search for robust biomarkers that can capture both current state and long-term effects of environmental exposures. Differences in welfare and behavioral strain characteristics have been documented in poultry, and previous work has shown that DNA methylation signatures can vary in relation to behavioral patterns in laying hens (Guerrero-Bosagna et al., 2020). Moreover, layer strains (e.g. brown versus white hens) differ in temperament, production traits, and propensity for abnormal behaviors, suggesting that genotype may modulate the impact of housing conditions on welfare (Dudde et al., 2018). In a recent study, differing DNA methylation signatures were reported in chicken breast muscle of 1-month-old chickens raised in different countries and continents (Venkatesh et al., 2023), further supporting the notion that environmental context and genetic background jointly shape epigenetic profiles.

Behavioral regulation in both humans and chickens involves several key neuroendocrine and neurotransmitter systems. In humans, adverse experiences during childhood and adolescence have repeatedly been associated with altered DNA methylation of the oxytocin receptor gene (Fujisawa et al., 2019; Muller et al., 2023). In chickens, mesotocin/oxytocin-family signaling has been linked to regulation of social behavior (Loveland et al., 2019). Similarly, human studies suggest that adverse experiences in childhood and adolescence can be associated with altered methylation of the serotonin system, including serotonin receptors, although findings are mixed and locus-specific (Beach et al., 2010; Goldenthal et al., 2024). Estrogen, acting through its receptor ESR1, has been identified as a key hormonal controller of social behavior in mammalians (reviewed by Lawal et al., 2025). In chickens, sex hormones, including estrogen, have been implicated in aggressive behavior in chickens (Lin et al., 2024). Based on this evidence, we focused on a set of candidate genes implicated in social behavior, stress responsiveness, and affective states: OXT, OXTR, ESR1, HTR1A, HTR2A, and HTR3A.

Environment and genotype can interact to shape DNA methylation, which is a key epigenetic mark involving the transfer of a methyl group onto the C5 position of cytosine to form 5-methylcytosine (Venkatesh et al., 2023). DNA methylation in turn can modulate gene expression, thereby affecting health and productivity amongst other traits, sometimes even across generations (McRae et al., 2014). Modulation of gene expression through methylation marks primarily occurs via recruiting proteins involved in gene repression or by inhibiting the binding of transcription factors, thereby exerting primarily a repressive effect on gene expression. DNA methylation can however also stimulate gene expression through, primarily via gene body methylation (Dhar et al., 2021). CpG sites (cytosine-phosphate-guanine dinucleotides) are underrepresented in the genomes of vertebrates, occurring at a frequency much lower than would be expected by random chance. This is attributed to the high mutation rate of methylated cytosines, which are prone to spontaneous deamination, causing a permanent cytosine to thymine transition. Unlike other dinucleotides, CpG sites frequently occur in clusters termed CpG islands, that are most commonly located near gene promoters (Sved and Bird, 1990). DNA methylation is increasingly recognized as a robust biomarker for animal welfare, as DNA methylation can provide a dynamic response to environmental cues and reflect long-term effects of environmental exposures.

We hypothesized that age, laying intensity, genetics, and/or housing shape methylation of genes implicated in behavior. The objective of the present study was therefore to compare DNA methylation signatures of selected genes implicated in behavior in chicken that were either housed in a layer cage or housed in small groups of 10 animals. To test the aforementioned hypothesis, the present study determined the effect of age (two-day-old chicks versus one-year-old laying hens), genetics (brown versus white layer hens), laying intensity (high versus low), and the effect of housing conditions (layer cage versus small group housing) on the methylation levels of the OXT, OXTR, ESR1, HTR1A, HTR2A, and HTR3A gene. Results demonstrate age, genetics, laying intensity, and housing conditions affecting DNA methylation of the oxytocin receptor gene in female chicken, while none of the other genes were consistently affected.

## Materials and methods

### Sample collection/Animal experiments

All procedures involving animals were carried out with the necessary approval by the local governmental authority. The collection of blood samples from chicks was approved for the scope of another project by the Lower Saxony State Office for Consumer Protection and Food Safety (LAVES; permit 33.19-42502-05-20A530). Housing of hens in a layer cage took place under a special agreement with the regulatory authorities (at the time of publication the housing set up in layer cages has been discontinued).

**Fig. 1:**
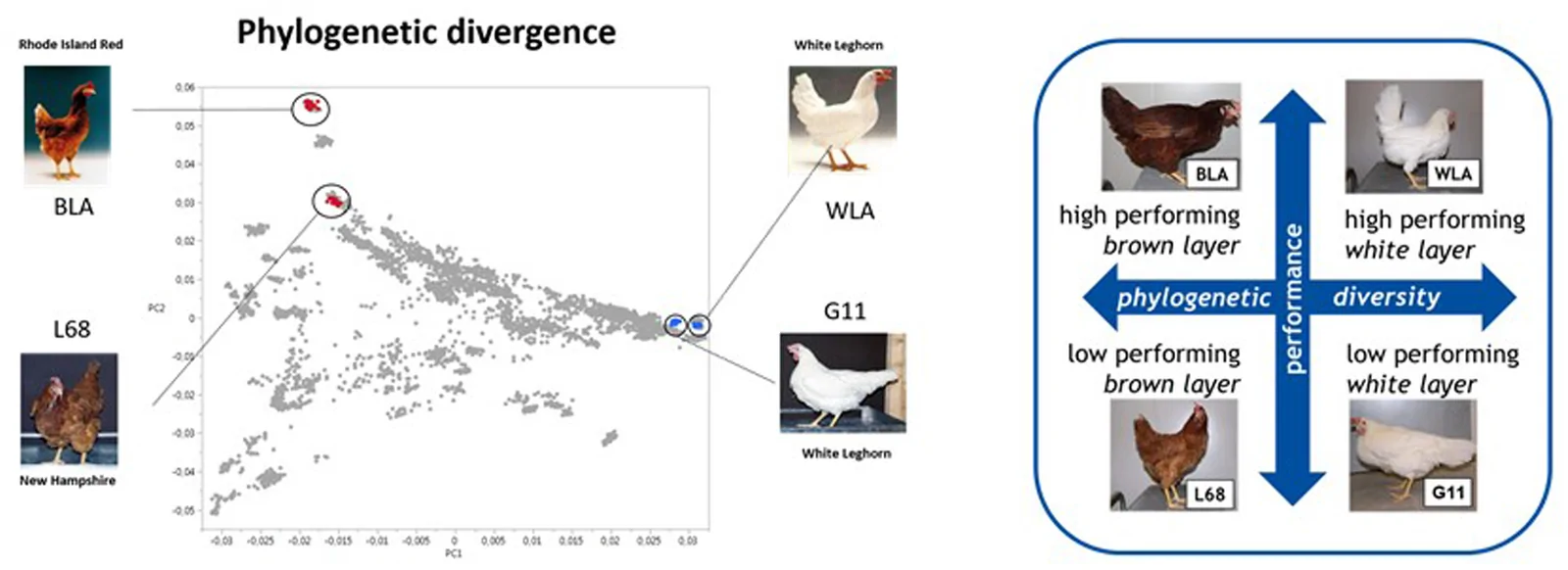
Divergence in phylogenetic origin and egg laying performance of the four purebred layer lines used in the present study.

Female animals from four purebred layer lines (*Gallus gallus domesticus*) differing in egg laying performance and phylogenetic origin in a cross-over design (Fig. 1) were used in the present study. The origin of these lines has been described in detail previously (Dudde et al., 2018; Malomane et al., 2021). L68 and BLA are brown layer lines characterized by low and high laying intensity respectively. G11 and WLA are white layer lines characterized by low and high laying intensity respectively. Low laying intensity amounts to around 200 eggs annually, while high laying intensity amounts to up to 320 – 340 eggs per year. Blood samples from 2-day old female chicks (n=6 per layer line) were collected via venipuncture within the scope of another experiment and surplus material was used for the present investigation. Blood samples from adult animals were collected at 454 ± 31 days of age at the time of slaughter (n=6 per layer line and housing condition). As chicks, animals were raised in groups of roughly 200 pullets (separated by line) in the same barn until the 16th week of age after which they were either housed in a layer cage under a special agreement with the regulatory authorities (one animal per compartment) or they were housed in small groups of 10 animals with access to group nests, perches, litter, pecking blocks and an additional sand tank for dustbathing. Adult animals were housed in the same barn, which contains 4 individual units (each 30 m x 6 m) for animal housing. Each units contained animals of two different layer lines, with 14 pens per layer line. Animals for analyses were sampled from random sites in the barn. The light program entailed an initial phase of 24 hours of light daily, to then decline from 15 hours of light daily in week 1 to 8 hours of light daily by week 9. Between weeks 18 -23, light was progressively extended again, reaching 14 hours of light daily by week 24. Barn temperature was reduced from 30–32°C in week 1 to approximately 18°C by week 5, then maintained near that level. Feed was provided ad libitum. Blood samples collected from chicks and adult animals were obtained from different individuals at each time point.

### Direct bisulfite sequencing

Blood samples from the two-day-old chickens were collected on filter paper cards for subsequent DNA extraction as described by Dierks and co-workers (Dierks et al., 2026). Blood from adult animals was collected into EDTA-coated tubes followed by centrifugation at 500 x g for 10 minutes to sediment red and white blood cells. DNA was then extracted from 5µl of (packed) red blood cells using the DNeasy Blood & Tissue Kit (Qiagen, Hilden, Germany) kit as per manufacturer’s instructions with an elution volume of 100µl. 300 ng of DNA were used as input for bisulfite treatment using the EZ DNA Methylation-Gold kit (Zymo Research, Irvine, CA, USA) following the manufacturer’s protocol, eluted in 25 µl elution buffer. Primers were designed using “Bisulfite Primer Seeker” (Zymo Research) and are listed in Table 1 (including position relative to translation start site). PCR reactions were carried out using 0.2µM of each primer, 200µM dNTP´s, 2 mM MgCl2, 1xPCR-buffer, and 2 Units Platinum™ Taq DNA-Polymerase (Invitrogen, Waltham, MA, USA). Following 94°C for 2min, 38 cycles of 20s at 94°C, 30s at 55°C, and 30s at 72°C were implemented. PCR reactions displaying a single band of expected size upon DNA gel electrophoresis were cleaned up using MSB® Spin PCRapace kit (Invitek Diagnostic, Berlin, Germany) and submitted to Sanger sequencing (Eurofins, Rostock, Germany). Resulting sequences were analyzed as described by Jiang and co-workers using Chromas (Informer Technologies, Inc., CA, USA) (Jiang, et al., 2010).

**Table 1:** **Primer sequences**. TSS = translation start site.

**Gene**	**Primer name**	**Accession number**	**forward primer**	**reverse primer**	**# CpG sites**	**position from TSS**	**Note**
**OXT**	OXT_1	XM_040671655.2	AGTTTGGTTATTTAAGGAGGTTT	AACACAAAACATTACTTTCCACA	6	-144 to -230	
	OXT_2		TGTGGAAAGTAATGTTTTGTGTT	CACAAAAACCTACCTTCCTAATA	10	+96 to -90	
**OXTR**	OXTR_1	NM_001031569.2	TTTAGYGGTGTTTTGGTAGTATT	CCTCAACTACRATTTTAAACTCTA	28	-537 to -713	0% methylation
	OXTR_2		YGGTTATGGAGAAGTTGTATTT	ACCTACAAATACTTAATCAACCR	24	+20 to +355	
**HTR1A**	1-HTR1A	NM_001170528.1	GTTGTTGTTGTTTTTYGGATAATT	ACRTAAATCTCCCTATAACCAAAT	25	-391 to -671	0% methylation
**HTR2A**	1-HTR2A	XM_046908587.1	GAAYGAGGTAGTGAGTAAGAAA	ACATCCTTCAACATTTAACCAA	19	-849 to -1136	0% methylation
	2-HTR2A		TGGGTTTTTAGTGTTAATTTTGG	CAAAACTCTCCTCATCACAAAA	9	-2 to -182	
**HTR3A**	1-HTR3A	XM_040690408.2	TGTGATGGGGAAATTTTTTATTG	ACCCAATCAAAAAAACAAACAA	12	-86 to -305	
	2-HTR3A		GTTGTTTGTTTTTTTGATTGGG	AAAAACACAAAAACAACCCTAA	15	-86 to +172	
**ESR1**	ESR1	NM_205183.2	GGTAGTGGATGGAGTTTATAGTT	CTCCACAACTACACTACTCTTC	6	-643 to -800	0% methylation

### Experimental design and statistical analyses

An initial screening for differences in DNA methylation levels was performed using samples from WLA and L68 obtained from 2-day old chicks and adult animals housed in cages using all primers (WLA and L68 display the largest differences with respect to genotype and laying intensity). Methylation levels between (1) young and old animals within each genotype and (2) between WLA and L68 adult animals were compared using t-tests followed by false discovery rate (FDR) correction to account for multiple testing. FDR correction was carried out for each gene individually. Since OXTR was the only gene displaying consistent differences in methylation levels in the initial screening, follow-up analyses were carried out targeting the OXTR gene only. Samples obtained from 2-day old chicks were subjected to analysis targeting the OXTR and a one-way ANOVA followed by FDR correction was used to determine differences in mean methylation levels between the four lines. Likewise, samples obtained from adult animals of all four layer lines housed under both conditions (cage and group housing) were subjected to analysis targeting the OXTR gene only. A three-way ANOVA followed by Tukey’s post hoc test was used to determine an interaction between genetics, housing condition and laying intensity on mean methylation levels of OXTR. A two-way ANOVA analysis followed by Tukey’s post hoc test was used to test for interaction between genetics and laying intensity for each housing condition separately. In both cases, FDR correction was implemented for the number of CpGs analyzed. A t-test followed by FDR correction was used to compare methylation of OXTR within each layer line for animals housed in a layer cage or housed in groups. Statistical analyses were performed using GraphPad Prism 10.

## Results

CpG sites analyzed for the primers OXTR_1, 1-HTR1A and 1-HTR2A displayed 0% methylation. Likewise, the first six CpG sites targeted by the primer OXTR_2 displayed 0% methylation. All remaining CpG sites that were analyzed displayed a varying level of methylation.

### Effect of age on DNA methylation

**Fig. 2:**
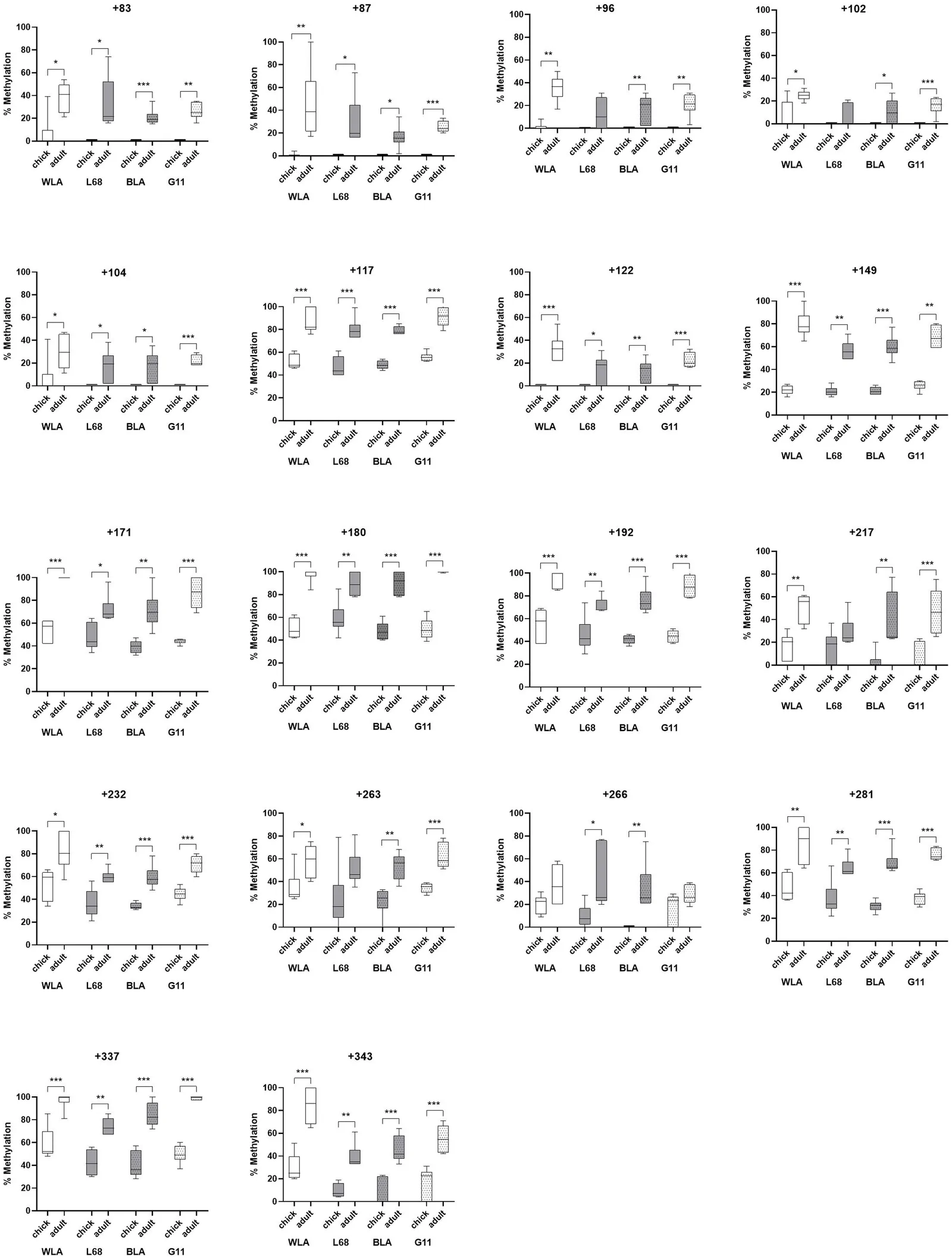
Methylation levels the OXTR gene of samples collected from WLA, L68, BLA, and G11 layer lines from 2-day old chicks and adult animals kept in layer cages. Superscripts indicate statistical significance (t-test followed by multiple testing correction (FDR-adjusted p-values (q)). *: q ≤ 0.05; **: q ≤ 0.01; ***: q ≤ 0.001).

No differences in methylation levels were detected for CpG sites analyzed for the HTR2A gene, while 10/18 (L68) and 2/18 (WLA) CpG sites displayed significant higher methylation levels in adult animals compared to 2-day old chickens for the HTR3A gene. Within the OXT gene, 2 of 17 CpG sites displayed significant differences between age for both L68 and WLA (supplementary Fig. 1). The greatest effect of age on mean methylation levels was detected for the OXTR gene (18 CpG sites located +83 to +343 of the translation start site). 17/18 (WLA), 14/18 (L68), 18/18 (BLA), and 17/18 (G11) CpG sites displayed significantly higher methylation levels in adult animals compared to 2-day old chicks (Fig. 2).

### Differences between adult L68 and WLA animals housed in layer cages

Eleven of eighteen CpG sites analyzed within the OXTR exhibited statistically significant differences in mean methylation, with L68 displaying a consistently lower methylation percentage than WLA (supplementary Fig. 2). None of the other genes analyzed showed a difference in methylation levels between adult L68 and WLA animals housed in layer cages.

### Differences between 2-day old chicks in OXTR methylation

One way-ANOVA followed by FDR correction revealed no significant differences in DNA methylation levels among the four layer lines at any of the 18 CpG sites interrogated for the OXTR gene.

### Effect of genetics, housing condition and laying intensity on OXTR methylation

**Fig. 3:**
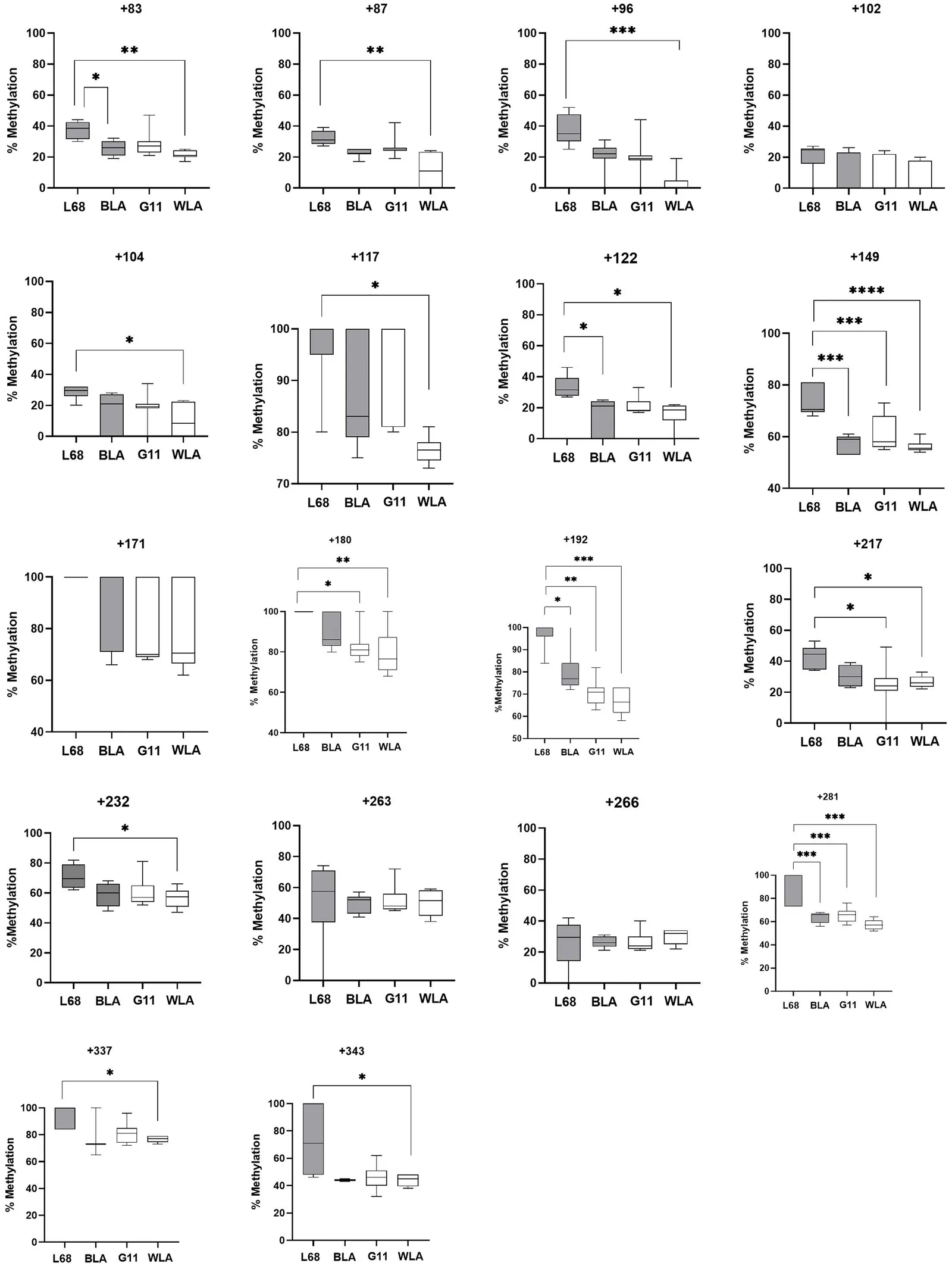
**Effect of genetics and laying intensity on OXTR methylation for adult chickens housed in groups.** CpG sites are named according to their distance from the translation start site. Two-way ANOVA analysis followed by Tukey’s post hoc test (FDR-adjusted p‑values (q)). *: q ≤ 0.05; **: q ≤ 0.01; ***: q ≤ 0.001.

**Fig. 4:**
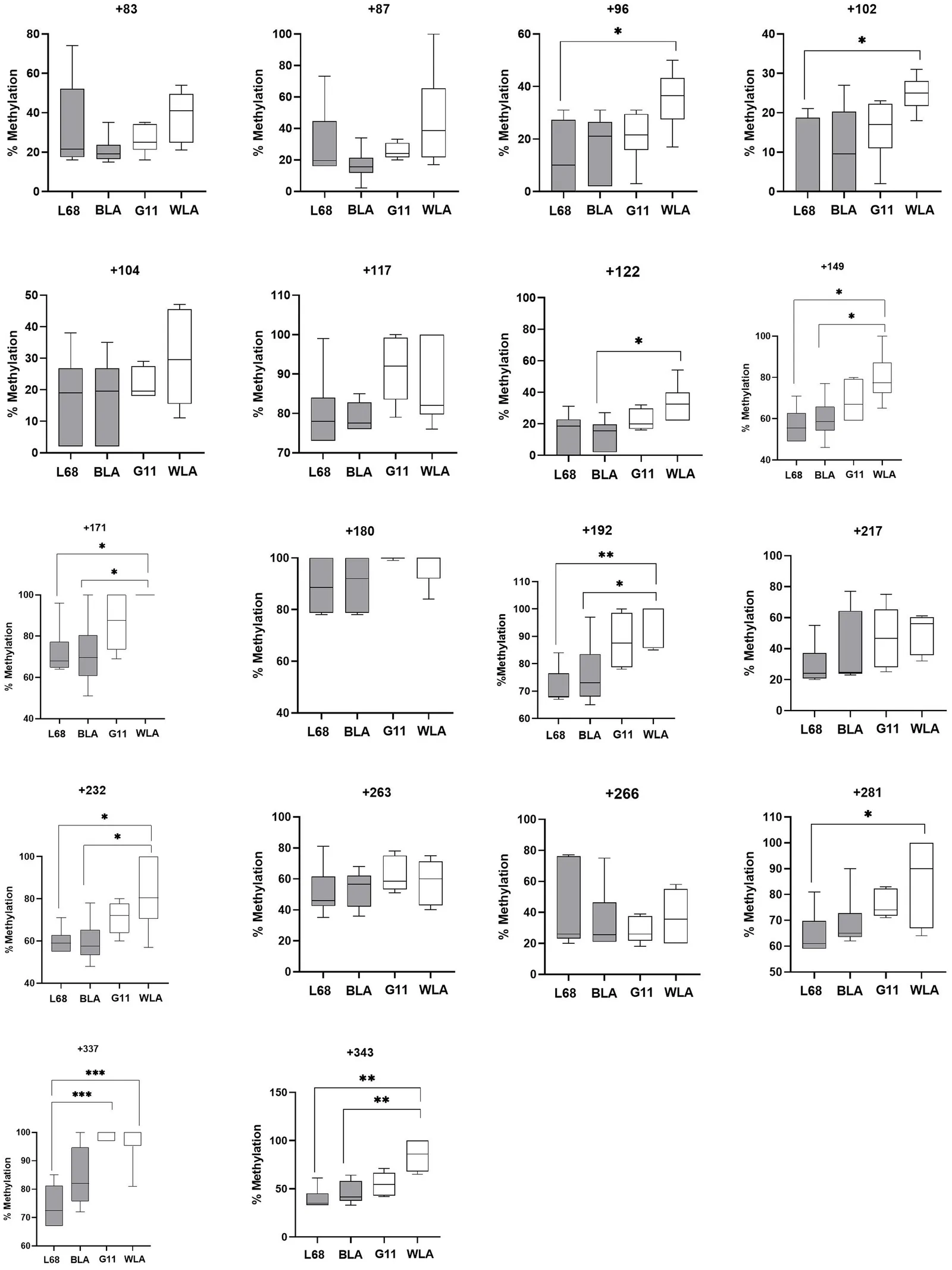
**Effect of genetics and laying intensity on OXTR methylation for adult chickens housed in layer cages.** CpG sites are named according to their distance from the translation start site. Two-way ANOVA analysis followed by Tukey’s post hoc test FDR-adjusted p‑values (q). *: q ≤ 0.05; **: q ≤ 0.01; ***: q ≤ 0.001.

Three-way ANOVA revealed a significant interaction effect of laying intensity and housing on 11/18 CpG sites and a significant interaction effect of genetics and housing on 15/18 CpG sites. For 4/18 CpG sites laying intensity x genetics affected methylation levels, while for 4/18 CpG sites a significant interaction effect of all three factors was present (Table 2). A follow up two-way ANOVA examined the effects of genetics and laying intensity for each housing condition. For samples obtained from animals housed in a layer cage, except for one CpG site, no effect of laying intensity on mean methylation levels was detected, while genetics had a significant impact on 12/18 CpG sites. For samples obtained from animals subjected to group housing, laying intensity had a significant effect on 12/18 CpG sites, and genetics had a significant effect on 11/18 CpG sites (Table 3, Fig. 3, Fig. 4).

**Table 2:** **Effect of genetics, housing condition, and laying intensity on OXTR methylation.** CpG sites are named according to their distance from the translation start site. Three-way ANOVA analysis followed by Tukey’s post hoc test (FDR-adjusted p‑values (q)). n.s. not significant.

**Table 3:** **Effect of genetics and laying intensity on OXTR methylation for adult chickens housed in a layer cage or group housed.** Two-way ANOVA analysis followed by Tukey’s post hoc test (FDR-adjusted p‑values (q)). n.s. not significant; *: q ≤ 0.05; **: q ≤ 0.01; ***: q ≤ 0.001; ****: q ≤ 0.0001.

**OXTR**	**layer cage**			**group housing**		
	**egg laying intesity**	**genetics**	**interaction**	**egg laying intesity**	**genetics**	**interaction**
**+83**	n.s.	n.s.	n.s.	***	*	n.s.
**+87**	n.s.	n.s.	n.s.	***	*	n.s.
**+96**	n.s.	*	n.s.	***	***	n.s.
**+102**	n.s.	**	n.s.	n.s.	n.s.	n.s.
**+104**	n.s.	n.s.	n.s.	*	*	n.s.
**+117**	n.s.	*	n.s.	***	n.s.	n.s.
**+122**	n.s.	**	n.s.	**	n.s.	n.s.
**+149**	n.s.	***	n.s.	****	**	**
**+171**	n.s.	***	n.s.	n.s.	*	n.s.
**+180**	n.s.	*	n.s.	n.s.	***	n.s.
**+192**	n.s.	***	n.s.	***	****	*
**+217**	n.s.	n.s.	n.s.	n.s.	**	n.s.
**+232**	n.s.	***	n.s.	*	n.s.	n.s.
**+263**	n.s.	n.s.	n.s.	n.s.	n.s.	n.s.
**+266**	n.s.	n.s.	n.s.	n.s.	n.s.	n.s.
**+281**	n.s.	**	n.s.	****	***	*
**+337**	n.s.	****	n.s.	*	n.s.	n.s.
**+343**	**	****	*	*	*	*

### Differences between group housed animals versus animals housed in a layer cage

**Fig. 5:**
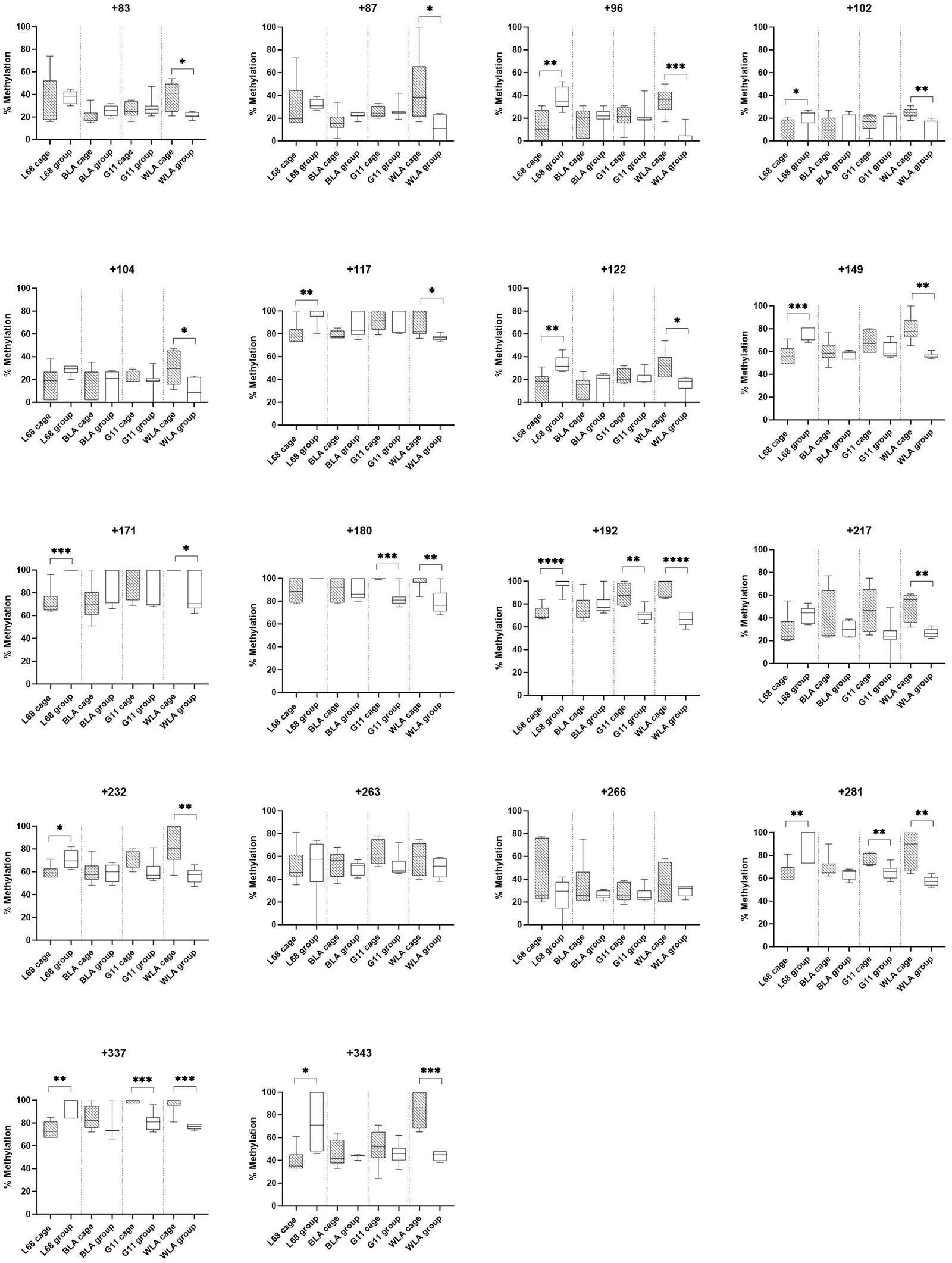
Methylation levels of OXTR in samples collected from adult animals of 4 layer lines housed in a layer cage or housed in groups. Asterisks indicate differences in mean methylation were between animals housed in a layer cage versus animals housed in groups within a given line (t-test followed by multiple testing correction (FDR-adjusted p‑values (q)). *: q ≤ 0.05; **: q ≤ 0.01; ***: q ≤ 0.001).

No significant differences for animals housed in groups compared to animals housed in a layer cage were detected for BLA, while for L68, 11/18 CpG sites displayed significantly higher levels of methylation for animals housed in group. Analysis of samples obtained from WLA, revealed significant differences for 16/18 CpG sites, with consistently higher methylation levels in samples obtained from animals housed in a layer cage. Similarly, 4/18 CpG sites displayed higher methylation levels in samples obtained from animals housed in a layer cage for G11 (Table 4, Fig. 5).

**Table 4:** Effect of housing condition OXTR methylation for adult chickens. Mean methylation were compared between animals housed in a layer cage versus housed in groups for each of the four layer lines CpG sites are named according to their distance from the translation start site. T-test followed by multiple testing correction (FDR-adjusted p‑values (q)). n.s. not significant; *: q ≤ 0.05; **: q ≤ 0.01; ***: q ≤ 0.001).

## Discussion

Environment and genotype can interact to shape DNA methylation. The present study evaluated the effects of genetics, laying intensity, and housing conditions on the methylation status of genes related to behavior. We utilized a unique animal model consisting of chickens from four purebred layer lines, differing in egg laying performance and phylogenetic origin in a cross-over design. L68 and BLA are brown layer lines characterized by low and high egg laying intensity respectively, while G11 and WLA are white layer lines characterized by low and high egg laying intensity respectively. The brown layer lines and the white layer lines form two clearly distinct phylogenetic lineages. Animals were subjected to drastically different housing conditions starting at the age of 16 weeks, with either being housed in a layer cage with one animal per compartment or in small groups of 10 animals until the age of approximately 14 months. Even though the expression of genes related to behavior is primarily relevant in brain tissue, we utilized blood as a surrogate, as a potential biomarker for animal welfare monitoring needs to be non-lethal and ideally allow continued monitoring. Several studies in humans have provided evidence that OXTR methylation in peripheral, i.e. non-brain tissue samples such as blood (Fujisawa et al., 2019) and saliva (Krol et al., 2019) is sensitive to environmental cues. While we examined DNA methylation at several behavioral related genes, OXTR was the only gene displaying consistent differences in DNA methylation depending on age, genetics, laying intensity, and housing. Adult animals of all 4 lines displayed significantly higher methylation than 2-day old chicks for almost all of the OXTR CpG sites interrogated. Six of the 18 OXTR CpG sites that exhibited methylation in samples obtained from adult animals were characterized by a complete absence of methylation in 2-day old chicks. This is likely attributable to the epigenetic reprogramming occurring during development of chicken primordial germ cells (PGCs), which entails a global demethylation during migration to the gonads (Yu et al., 2019). However, unlike mammalian PGCs, chicken PGCs do not experience near-complete genome-wide DNA demethylation; instead, they retain substantial DNA methylation (Kress et al., 2024). This might explain why OXTR was the only gene displaying consistent differences in DNA methylation depending on age; for the remaining genes examined either none or few CpG sites were characterized by lower methylation values in young animals.

While OXTR methylation levels did not differ between 2-day old chicks, clear differences in OXTR methylation levels were detected for adult animals. Dissecting the influence of genetics, housing, and laying intensity revealed the majority of CpG sites being influenced by the interaction of genetics x housing and the interaction of laying intensity x housing. Evaluating animals housed in layer cages and animals housed in groups separately provided further insights into the effects of genetics and laying intensity. For animals housed in layer cages, 12/18 CpG sites were influenced by genetics, while laying intensity had no effect. This was reflected in the observation of both white lines exhibiting higher methylation levels at the majority (WLA), respectively selected (G11) CpG sites. For group housed animals on the other hand, the majority of CpG sites were influenced by both laying intensity and genetics, which is reflected by the increased methylation observed for the majority of CpG sites for L68 animals. Interestingly, samples collected from adult BLA animals did not display any differences between animals housed in groups or layer cages.

Reduced representation bisulfite sequencing revealed differences in DNA methylation profiles in jejunal mucosa of laying hens from two different laying hen strains at various ages (Lohmann Brown-Classic and Lohmann Selected Leghorn-Classic) (Ponsuksili et al., 2025). Based on the PCA plot, a clear group separation based on the genotype was evident, with the first principal component accounting for 32% of variation. These two laying hen strains have a similar phylogenetic divergence as the white and brown layer lines utilized in the present study (Malomane et al., 2019), supporting our finding that genetics has a major impact on DNA methylation. Genetic variants that influence DNA methylation levels are known as methylation quantitative trait loci (meQTLs), occurring both as cis- and trans-meQTLs. In chickens, meQTLs have been mapped using a wild x domestic intercross, revealing thousands of cis- and trans-acting loci controlling DNA methylation (Hoglund et al., 2020).

The present study relied on blood samples as a source for DNA. In chicken, methylated DNA Immunoprecipitation of DNA extracted from red blood cells from 24-week-old hens that were subjected to differing housing conditions revealed DNA methylation profiles to correlate housing conditions. Based on the PCA plot, a clear group separation based on rearing conditions was evident, with the first principal component accounting for 53% of variation (Pertille et al. 2017). The impact of environmental cues in the form of cage housing versus group housing on DNA methylation is confirmed by our observation of the majority of OXTR CpG sites being influenced by the interaction of genetics x housing and the interaction of laying intensity x housing. Similar to observations made in human, the differentially methylated region is located within the gene body, rather than the promoter region (Muller et al., 2023).

Oxytocin-family signaling (mesotocin acting via OXTR) in chickens has been linked to behavioral traits (mesotocin is the equivalent of mammalian oxytocin, differing only in one amino acid). Intracerebroventricular injection of oxytocin increased locomotor activity, while decreasing feed intake and preening in broiler chickens (Jonaidi et al., 2003). Mesotocin may also be relevant for social predisposition, as intracranial mesotocin injections led to higher hen preference scores, i.e. the tendency to approach a hen-like stimulus (Loveland et al., 2019). Replacing eggs with chicks in native Thai hens led to an increase in the number of mesotocin-immunoreactive neurons in several hypothalamic nuclei (Sinpru et al., 2018). Given the link of oxytocin/mesotocin signaling to behavioral traits and our findings that age, genetics, laying intensity, and housing conditions affect DNA methylation of OXTR in female chickens, the epigenetic status of OXTR is a potential biomarker in the context of animal welfare monitoring. Future studies will have to include welfare-related, behavioral, or physiological stress indicators, to confirm OXTR methylation as marker for welfare assessment in laying hens, or whether OXTR methylation is only connected to differences in cognition, emotion, and/or behavior.

Limitations of the study were the locus-specific bisulfite approach that focused on selected genes only. Nevertheless, the results point toward the usefulness of OXTR as a possible biomarker. Future work should combine whole genome methylation profiling with behavioral phenotyping, potentially extending the list of candidate biomarkers. A further limitation is the use of different pre-processing methods for samples from chicks and adult animals. We have previously validated the suitability of both pre-processing methods for methylation analyses with minimal effect on methylation results, although the OXTR gene had not included in the analyses. Therefore, the introduction of a potential technical bias in the comparison of samples from chicks and adult animals cannot be excluded. For obvious reasons, blood samples were used as a surrogate, even though OXTR expression relevant to behavior is primarily in the brain. Studies in humans have examined DNA methylation in blood and corresponding brain samples, revealing a positive correlation between methylation statuses for a considerable number of CpG sites interrogated for OXTR (Edgar, et al., 2017). However, the use of OXTR methylation of peripheral samples as a surrogate for brain epigenetic status is unvalidated in chickens.

## Author Contribution

Conceptualization: C. Klein, S. Weigend, C. Reimer. Methodology: C. Klein, S. Weigend, C. Reimer Formal analysis: C. Klein, C. Reimer. Investigation: C. Klein. Resources: A. Smith. Data Curation: C. Klein. Writing - Original Draft: C. Klein. Writing - Review & Editing: C. Klein, S. Weigend, C. Reimer. Visualization: C. Klein. Supervision: A. Smith. Project administration: C. Klein.

## Disclosures

The authors declare that they have no known competing financial interests or personal relationships that could have appeared to influence the work reported in this paper.

## References

[bib0001] Baxter, M. R. 1994. The welfare problems of laying hens in battery cages. Vet. Rec. 134:614-619. 0.1136/vr.134.24.614. 10.1136/vr.134.24.614 7941260

[bib0002] Beach S.R.H., Brody G.H., Todorov A.A., Gunter T.D., Philibert R.A. (2010). Methylation at SLC6A4 is linked to family history of child abuse: an examination of the Iowa Adoptee sample. Am. J. Med. Genet. B Neuropsychiatr. Genet..

[bib0003] Dhar G.A., Saha S., Mitra P., Chaudhuri R.Nag (2021). DNA methylation and regulation of gene expression: Guardian of our health. Nucleus. (Calcutta).

[bib0004] Dierks C., Forster A., Meunier D., Preisinger R., Klein C., Weigend S., Altgilbers S. (2026). In ovo sexing and genotyping using PCR techniques: a contribution to the 3R principles in chicken breeding. Sci. Rep..

[bib0005] Dudde A., Krause E.T., Matthews L.R., Schrader L. (2018). More than eggs - relationship between productivity and learning in laying hens. Front. Psychol..

[bib0006] Edgar R.D., Jones M.J., Meaney M.J., Turecki G., Kobor M.S. (2017). BECon: a tool for interpreting DNA methylation findings from blood in the context of brain. Transl. Psychiatry.

[bib0007] Fujisawa T.X., Nishitani S., Takiguchi S., Shimada K., Smith A.K., Tomoda A. (2019). Oxytocin receptor DNA methylation and alterations of brain volumes in maltreated children. Neuropsychopharmacology.

[bib0008] Goldenthal A.R., Lieberman E., Rizk M.M., Ogden R.T., Rubin-Falcone H., Zanderigo F., Huang Y.Y., Min E., Yuan M., Milak M., Sullivan G.M., Sublette M.E., Oquendo M.A., Mann J.J., Miller J.M. (2024). Relationships between serotonin 1A receptor DNA methylation, self-reported history of childhood abuse and gray matter volume in major depression. J. Affect. Disord..

[bib0009] Guerrero-Bosagna C., Pertille F., Gomez Y., Rezaei S., Gebhardt-Henrich S.G., Vogeli S., Stratmann A., Voelkl B., Toscano M.J. (2020). DNA methylation variation in the brain of laying hens in relation to differential behavioral patterns. Comp. Biochem. Physiol. Part D. Genomics. Proteomics..

[bib0010] Grethen K.J., Gomez Y., Toscano M.J. (2023). Coup in the coop: Rank changes in chicken dominance hierarchies over maturation. Behav. Processes..

[bib0011] Hoglund A., Henriksen R., Fogelholm J., Churcher A.M., Guerrero-Bosagna C.M., Martinez-Barrio A., Johnsson M., Jensen P., Wright D. (2020). The methylation landscape and its role in domestication and gene regulation in the chicken. Nat. Ecol. Evol..

[bib0012] Jiang M., Zhang Y., Fei J., Chang X., Fan W., Qian X., Zhang T., Lu D. (2010). Rapid quantification of DNA methylation by measuring relative peak heights in direct bisulfite-PCR sequencing traces. Lab. Invest..

[bib0013] Jonaidi H., Oloumi M.M., Denbow D.M. (2003). Behavioral effects of intracerebroventricular injection of oxytocin in birds. Physiol. Behav..

[bib0014] Kress C., Jouneau L., Pain B. (2024). Reinforcement of repressive marks in the chicken primordial germ cell epigenetic signature: divergence from basal state resetting in mammals. Epigenetics Chromatin..

[bib0015] Krol K.M., Moulder R.G., Lillard T.S., Grossmann T., Connelly J.J. (2019). Epigenetic dynamics in infancy and the impact of maternal engagement. Sci. Adv..

[bib0016] Lawal O.O., Lin D., Lischinsky J.E. (2025). Estrogen control of social behaviors. Annu Rev. Neurosci..

[bib0017] Lin J.C., Daigle C.L., Tang P.C., Wang C.K. (2024). Influence of sex hormones on the aggressive behavior during peck order establishment and stabilization in meat and egg type chickens. Poult. Sci..

[bib0018] Loveland J.L., Stewart M.G., Vallortigara G. (2019). Effects of oxytocin-family peptides and substance P on locomotor activity and filial preferences in visually naive chicks. Eur. J. Neurosci..

[bib0019] Malomane D.K., Simianer H., Weigend A., Reimer C., Schmitt A.O., Weigend S. (2019). The SYNBREED chicken diversity panel: a global resource to assess chicken diversity at high genomic resolution. BMC. Genomics..

[bib0020] Malomane D.K., Weigend S., Schmitt A.O., Weigend A., Reimer C., Simianer H. (2021). Genetic diversity in global chicken breeds in relation to their genetic distances to wild populations. Genet. Sel. Evol..

[bib0021] McRae A.F., Powell J.E., Henders A.K., Bowdler L., Hemani G., Shah S., Painter J.N., Martin N.G., Visscher P.M., Montgomery G.W. (2014). Contribution of genetic variation to transgenerational inheritance of DNA methylation. Genome Biol..

[bib0022] Muller S., Sicorello M., Moser D., Frach L., Limberg A., Gumpp A.M., Ramo-Fernandez L., Kohler-Dauner F., Fegert J.M., Waller C., Kumsta R., Kolassa I.T. (2023). The DNA methylation landscape of the human oxytocin receptor gene (OXTR): data-driven clusters and their relation to gene expression and childhood adversity. Transl. Psychiatry.

[bib0023] Pertille F., Brantsaeter M., Nordgreen J., Coutinho L.L., Janczak A.M., Jensen P., Guerrero-Bosagna C. (2017). DNA methylation profiles in red blood cells of adult hens correlate with their rearing conditions. J. Exp. Biol..

[bib0024] Ponsuksili S., Hadlich F., Li S., Trakooljul N., Reyer H., Oster M., Abitew Y.A., Sommerfeld V., Rodehutscord M., Wimmers K. (2025). DNA methylation dynamics in the small intestine of egg-selected laying hens along egg production stages. Physiol. Genomics..

[bib0025] Sinpru P., Sartsoongnoen N., Rozenboim I., Porter T.E., El Halawani M.E., Chaiseha Y. (2018). The effects of replacing eggs with chicks on mesotocin, dopamine, and prolactin in the native Thai hen. Gen. Comp. Endocrinol..

[bib0026] Sved J., Bird A. (1990). The expected equilibrium of the CpG dinucleotide in vertebrate genomes under a mutation model. Proc. Natl. Acad. Sci. u S. a.

[bib0027] Venkatesh G., Tonges S., Hanna K., Ng Y.L., Whelan R., Andriantsoa R., Lingenberg A., Roy S., Nagarajan S., Fong S., Raddatz G., Bohl F., Lyko F. (2023). Context-dependent DNA methylation signatures in animal livestock. Environ. Epigenet..

[bib0028] Yu M., Li D., Cao W., Chen X., Du W. (2019). Effects of ten-eleven translocation 1 (Tet1) on DNA methylation and gene expression in chicken primordial germ cells. Reprod. Fertil. Dev..

