# Supplementary material for: Variation in oxytocin receptor DNA methylation in female chickens are associated with age, genetics, laying intensity, and housing conditions
Source: Poult Sci. 2026 Aug 20;105(12):107667. doi: 10.1016/j.psj.2026.107667 (PMC13594890; doi:10.1016/j.psj.2026.107667)

Supplementary Figure 1: Effect of age on DNA methylation – OXT1

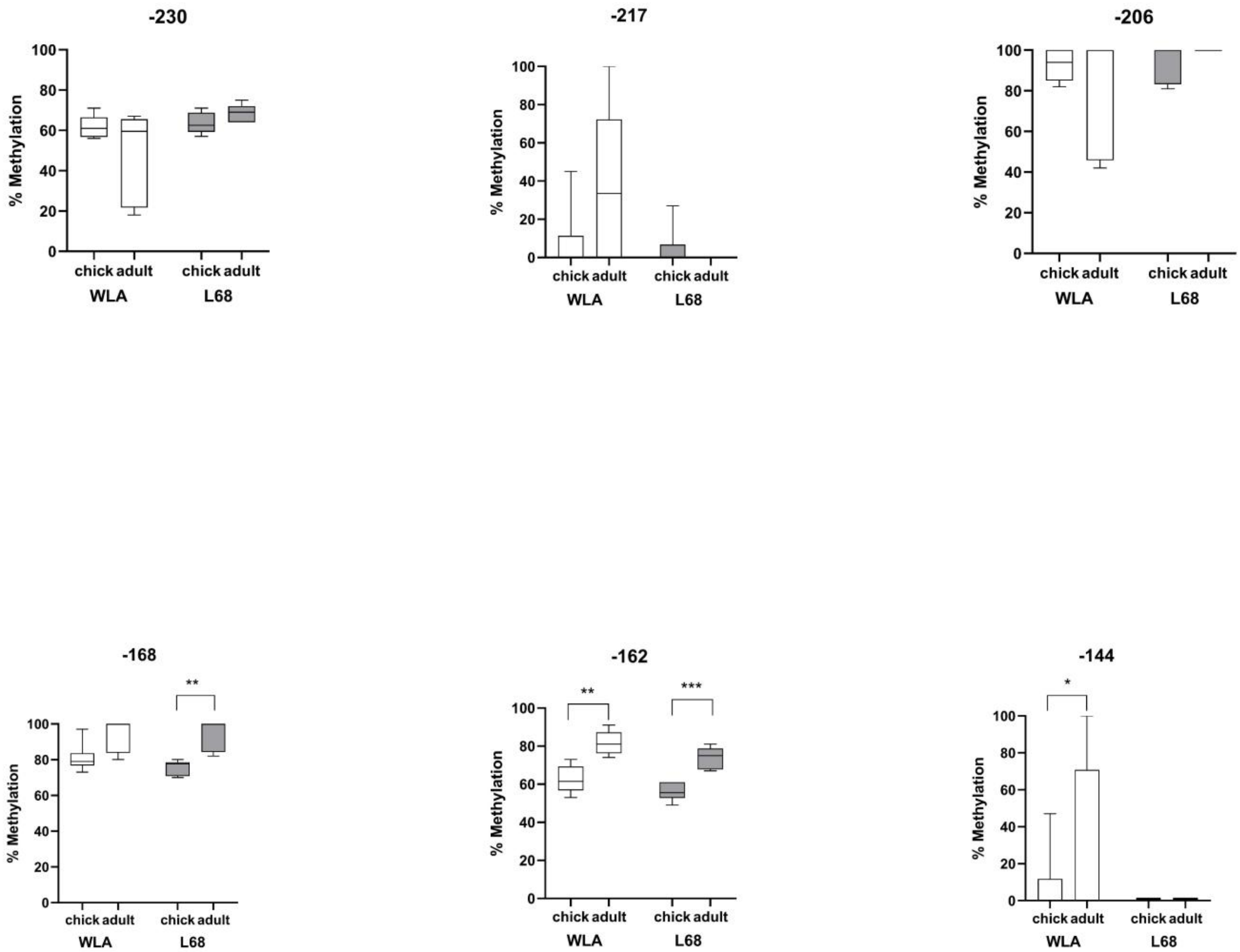

Supplementary Figure 1: Effect of age on DNA methylation – OXT2

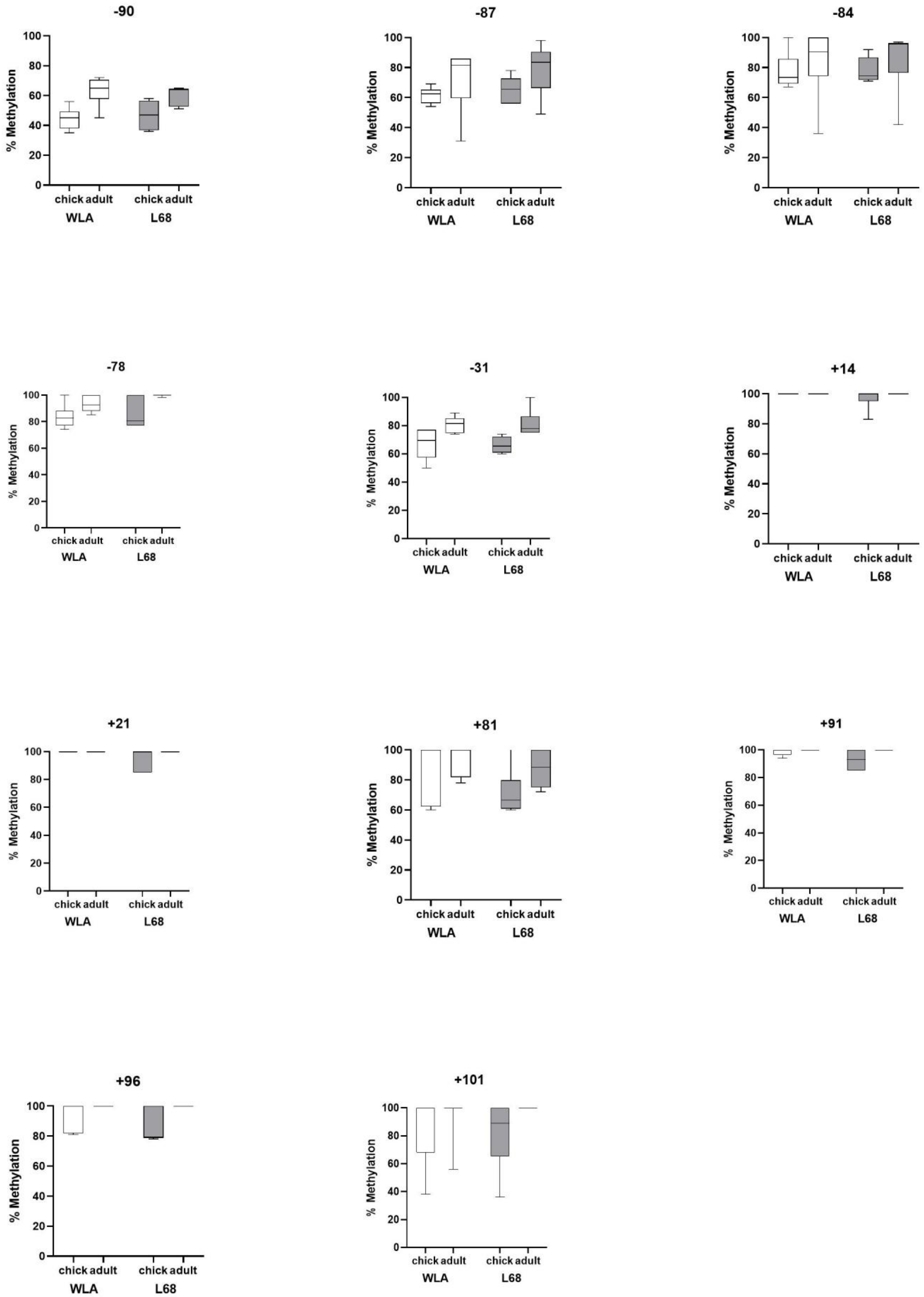

Supplementary Figure 1: Effect of age on DNA methylation – 1-HTR3A

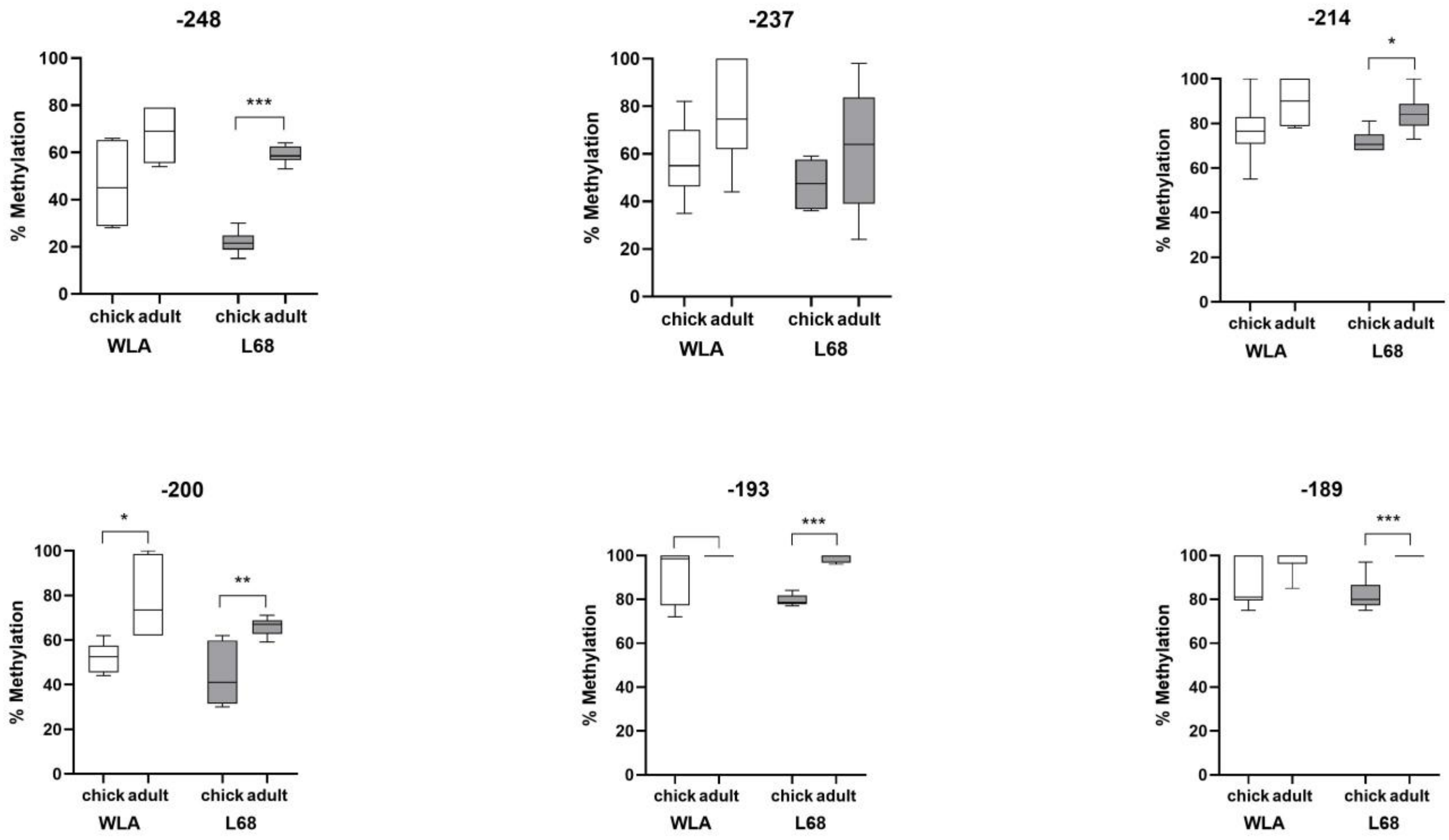

Supplementary Figure 1: Effect of age on DNA methylation – 2-HTR3A

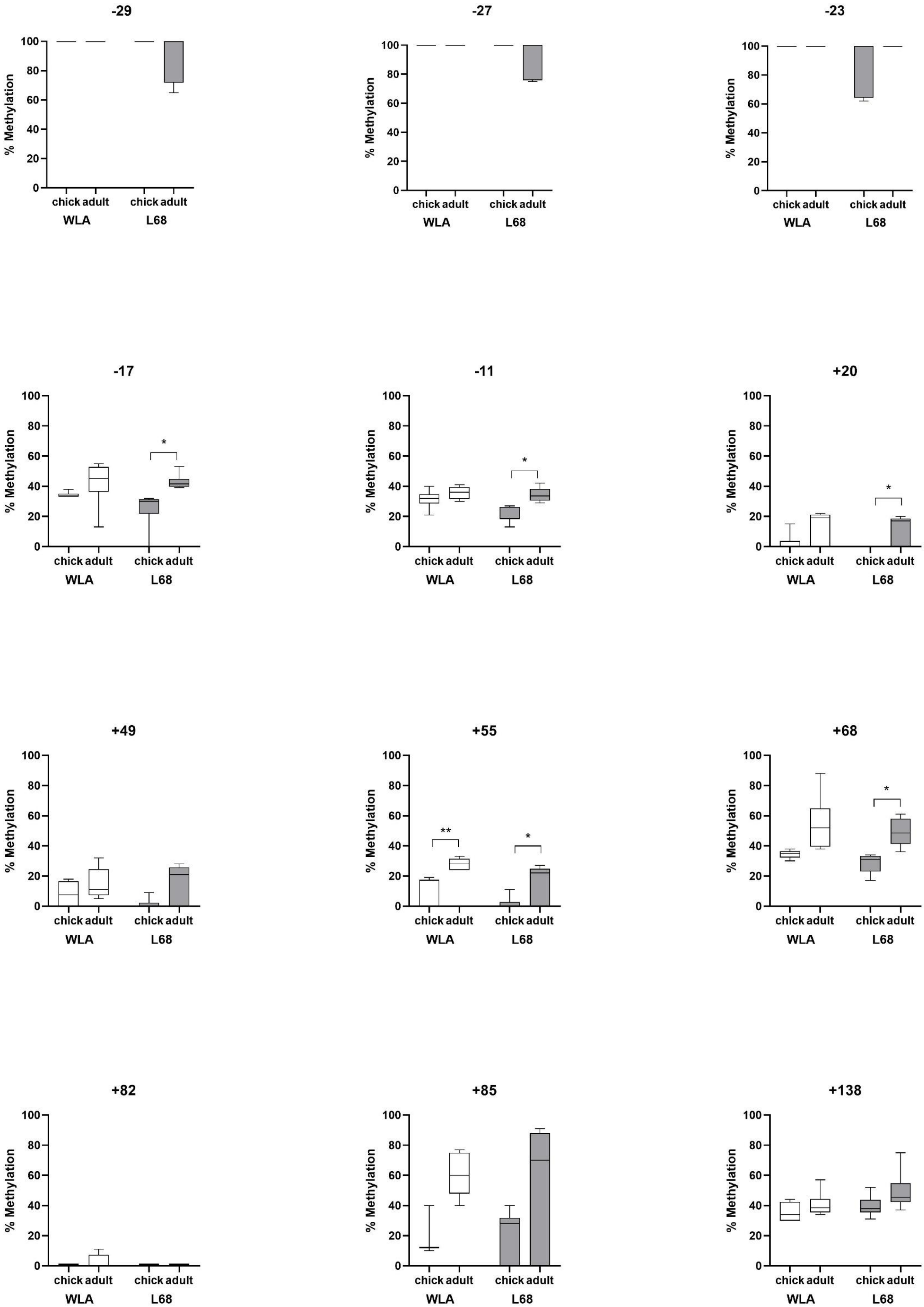

Supplementary Figure 1: Effect of age on DNA methylation – 2-HTR2A

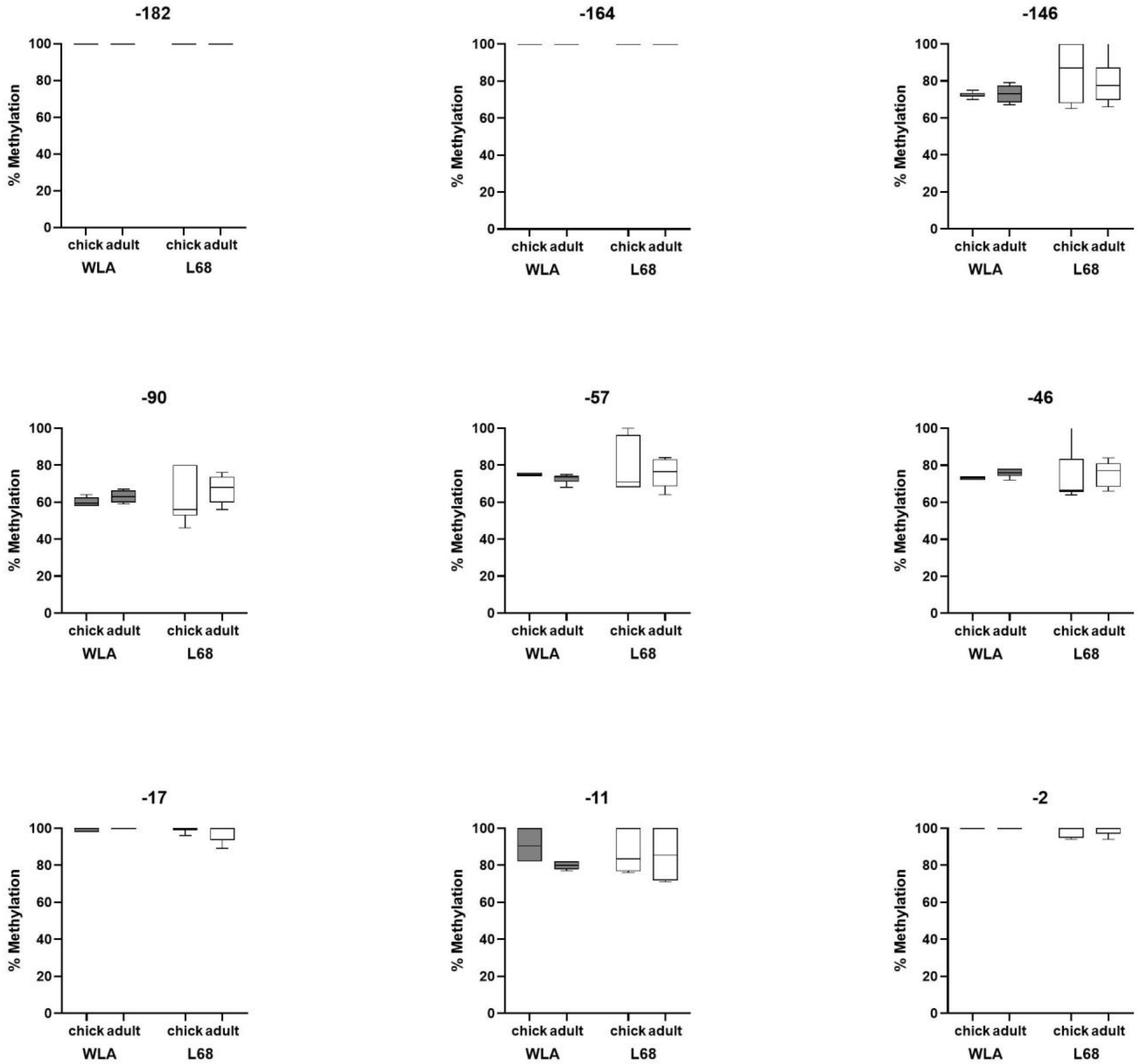

Supplement: Supplementary file 1 [file mmc1.pdf]
