# Supplementary material for: Variation in oxytocin receptor DNA methylation in female chickens are associated with age, genetics, laying intensity, and housing conditions
Source: Poult Sci. 2026 Aug 20;105(12):107667. doi: 10.1016/j.psj.2026.107667 (PMC13594890; doi:10.1016/j.psj.2026.107667)

Supplementary Figure 2:

Differences between adult L68 and WLA animals housed in layer cages – OXT1

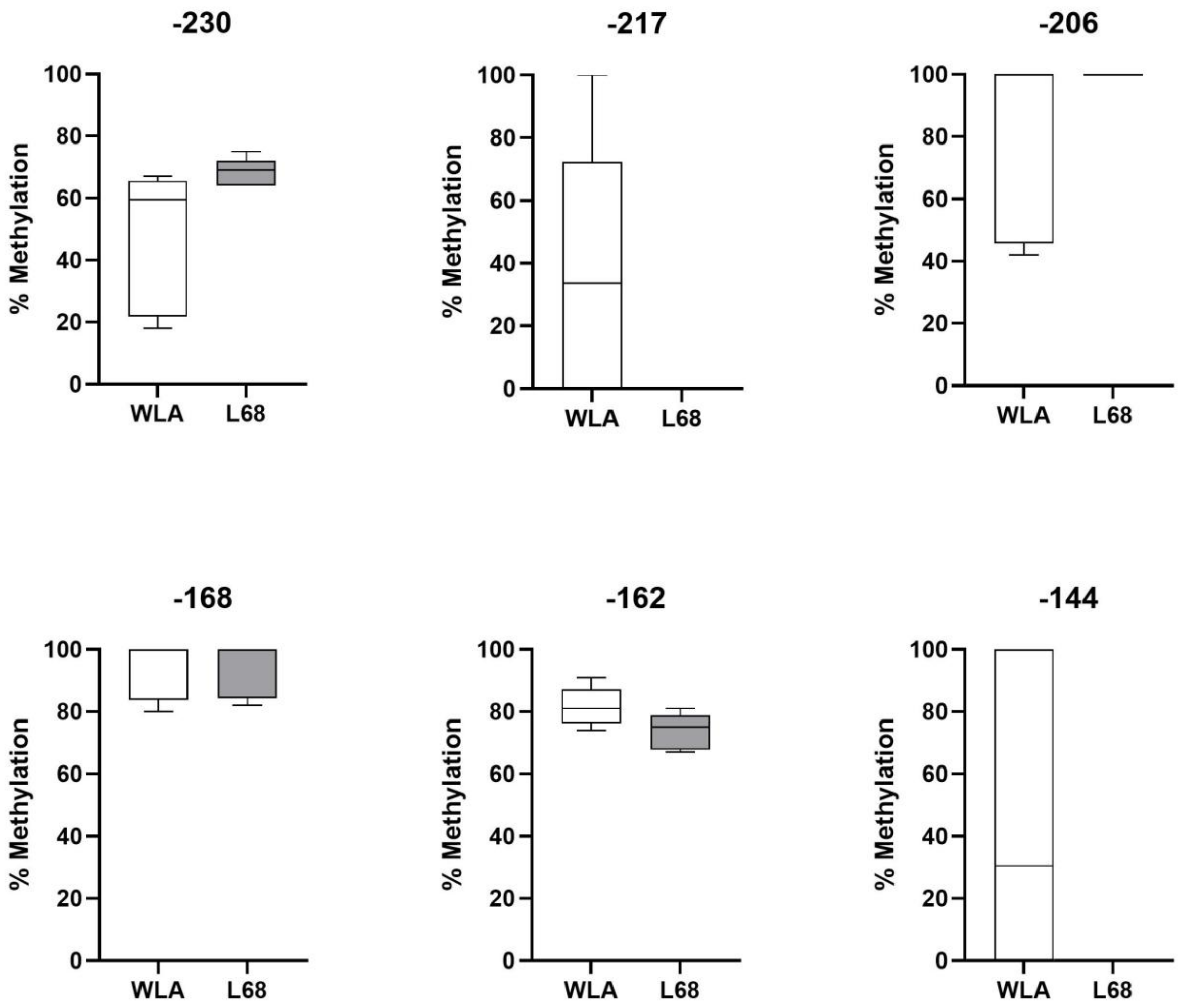

Supplementary Figure 2:

Differences between adult L68 and WLA animals housed in layer cages – OXT2

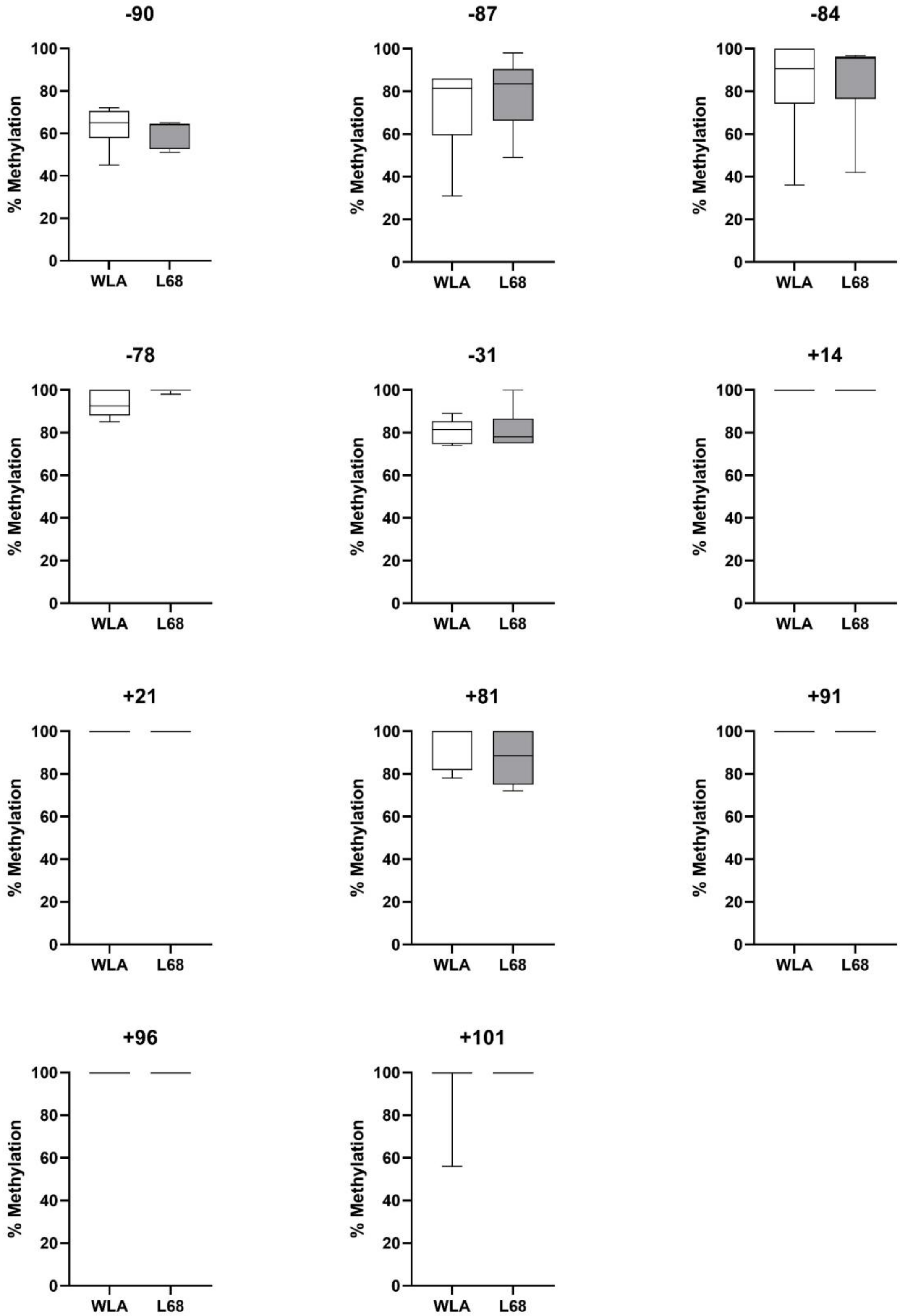

Supplementary Figure 2:

Differences between adult L68 and WLA animals housed in layer cages – 1-HTR3A

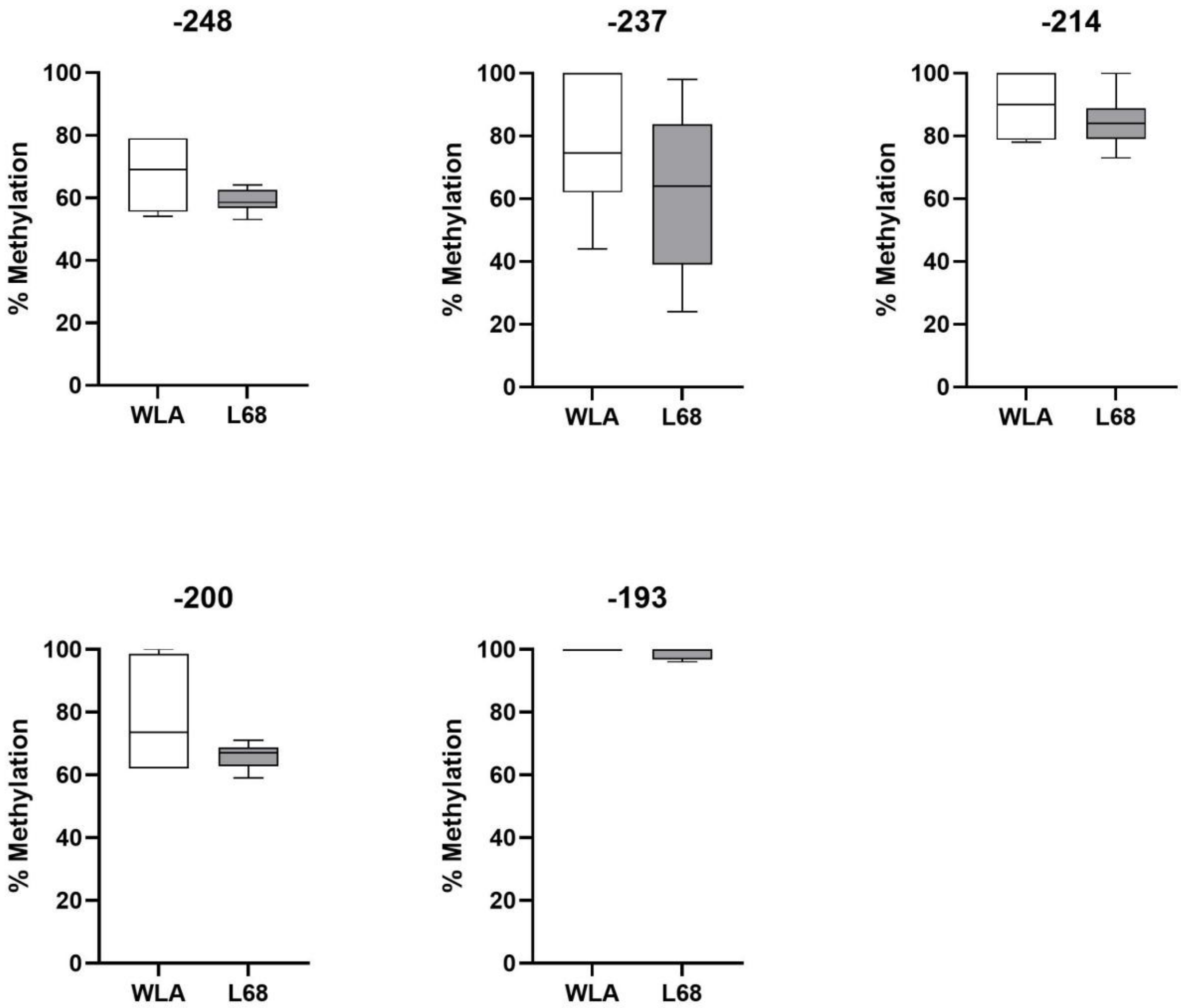

Supplementary Figure 2:

Differences between adult L68 and WLA animals housed in layer cages – 2-HTR3A

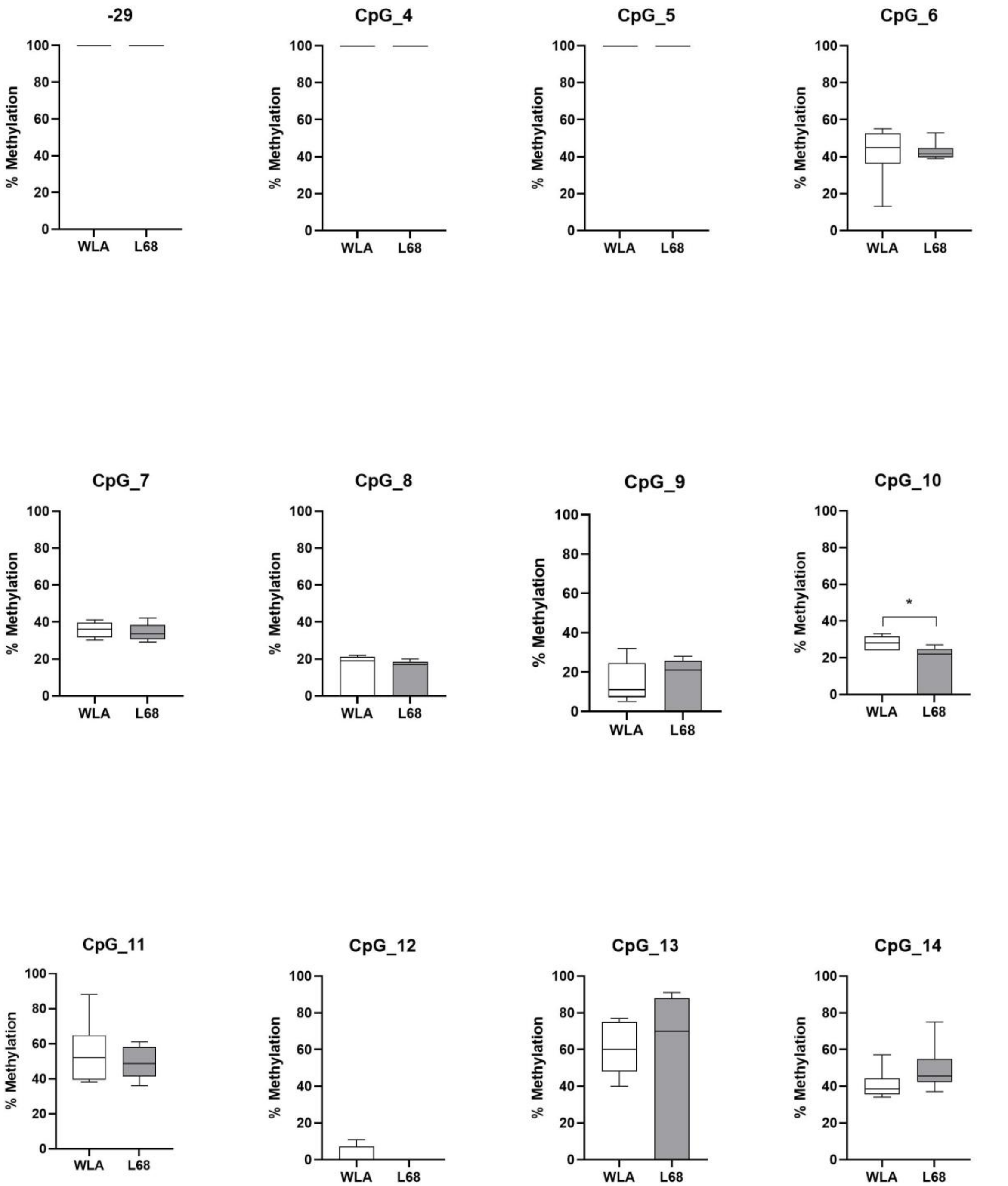

Supplementary Figure 2:

Differences between adult L68 and WLA animals housed in layer cages – 2-HTR2A

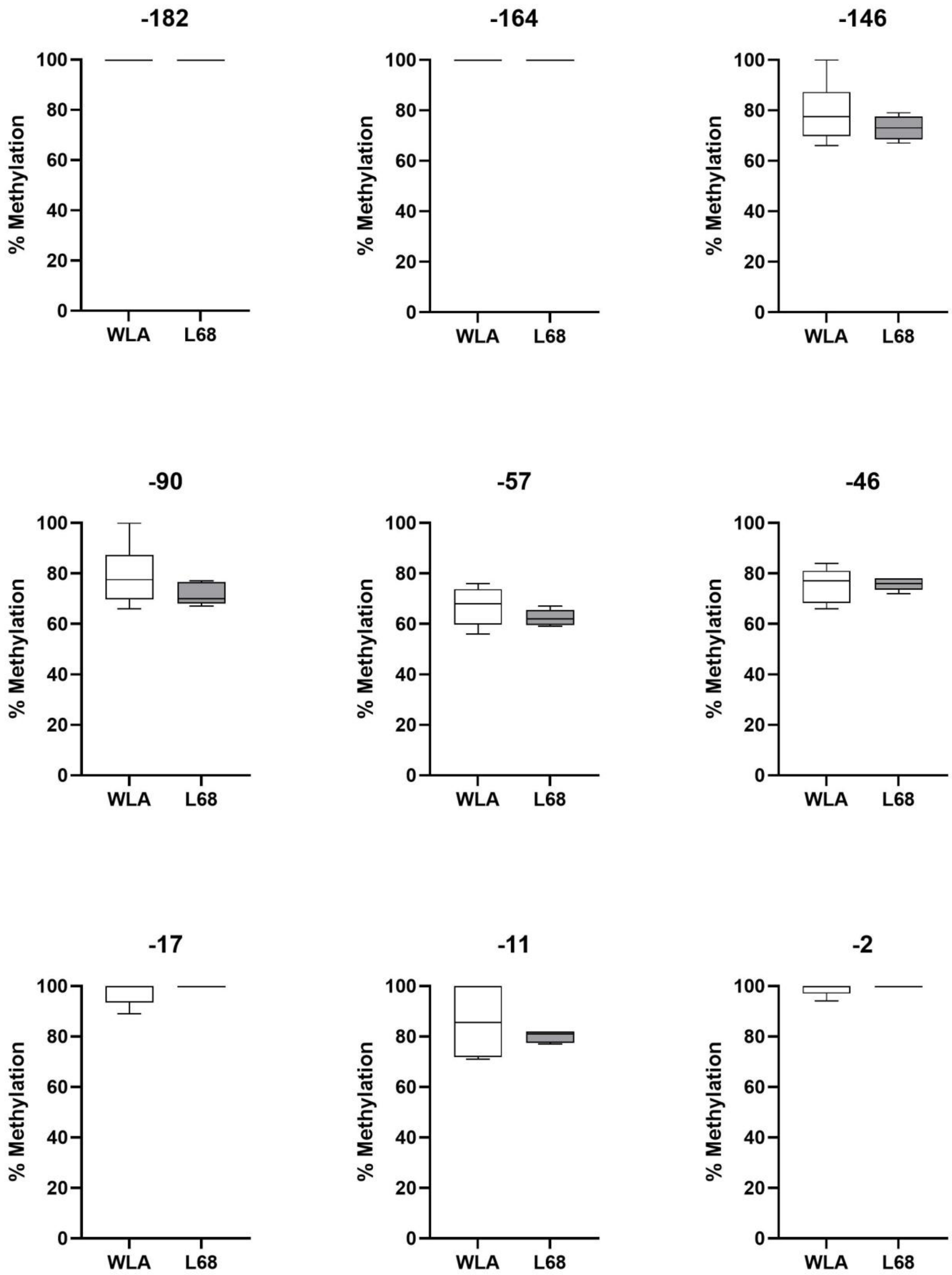

Supplement: Supplementary file 2 [file mmc2.pdf]
